# Artificial neural network predictive models for optimizing the training process in race walking: a longitudinal observational study

**DOI:** 10.7717/peerj.21224

**Published:** 2026-05-22

**Authors:** Dariusz Skalski, Magdalena Prończuk, Kinga Łosińska, Petr Stastny, Adam Maszczyk, Adam Zajac

**Affiliations:** 1 Gdańsk University of Physical Education and Sport, Gdansk, Poland; 2 Department of Sport Games, Faculty of Physical Education and Sport, Charles University in Prague, Prague, Czech Republic; 3 Institute of Sport Science, The Jerzy Kukuczka Academy of Physical Education, Katowice, Poland

**Keywords:** Race walking performance, Machine learning, Training adaptation, Physiological monitoring

## Abstract

This study identified key physiological, biomechanical, and strength-related predictors of competitive performance in elite female race walkers and evaluated the effectiveness of classical and machine-learning models for individualized training optimization. Thirty nationally ranked female race walkers (25 ± 3 years) were assessed over four seasons (2021–2024). Laboratory and field tests included ergospirometry (VO_2_max, VCO2, VE (minute ventilation), respiratory exchange ratio (RER)), blood lactate (LA), heart rate (HR), gait kinematics (step length, speed), and lower-limb strength (1RM, maximal power). Temporal and seasonal dynamics were evaluated using Kruskal–Wallis and g-Fisher tests. Predictive models included multiple regression, polynomial regression, multilayer perceptron (MLP), and radial basis function (RBF) networks, developed with correlation-vector analysis (R0, R1), collinearity diagnostics, and interaction terms. The most influential predictors were HR, step length, VO_2_max, and 1RM (R0 > 0.70). RBF achieved the best predictive accuracy (*R*^2^ = 0.89; RMSE = 0.28), outperforming MLP (*R*^2^ = 0.87) and regression baselines (*R*^2^ = 0.61–0.81). Significant seasonal variation (*p* < 0.001) underscored the value of time-dependent modeling. Conclusion: RBF neural networks offer superior performance for predicting race-walking outcomes; HR and step length are key real-time indicators, whereas VO_2_max and 1RM inform longer-term adaptation.

## Introduction

Race walking is among the most demanding and technically specific disciplines in track and field athletics (Sovenko et al., 2025). It is governed by unique technical rules and imposes high physical conditioning requirements (Bernans & Saulgriezis, 2023). As an endurance-based sport, race walking obligates athletes to maintain continuous ground contact with at least one foot and to fully extend the knee of the leading leg from the moment it contacts the ground until it reaches the upright vertical position (International Association of Athletics Federations, 2011). These strict technical constraints distinguish race walking from other running events, necessitating not only excellent physical conditioning but also a high degree of technical precision, both of which are critical to performance optimization (Murray et al., 1983; Hamdan et al., 2024).

The rapid development of technology and analytical methods has opened new avenues for monitoring and optimizing the training process in race walking (Bourdon et al., 2017). The integration of modern research tools such as biomechanical analysis, mathematical modeling, and artificial intelligence (AI) enables more accurate assessments of training effectiveness and allows for predicting athletes’ adaptive responses to various training loads (Wang, Wang & Sun, 2025). Enhanced motion analysis techniques, including motion capture systems and machine learning algorithms, now facilitate detailed tracking of technical parameters in race walking, such as step length, stride frequency, and joint angles during specific phases of the gait cycle (Hanley, Bissas & Drake, 2013). Recent advances in machine learning applications have demonstrated the potential of data-driven methods to improve athlete monitoring, personalized training, and performance prediction (Wang, Wang & Sun, 2025; Zhang et al., 2024; Li et al., 2025a; Li et al., 2025b).

Contemporary approaches to training optimization are increasingly based on systems thinking and control theory, which allow for the integration of various training components, physiological, biomechanical, and psychological (Bourdon et al., 2017). Systems analysis supports the modeling and prediction of athletic performance by incorporating key training variables such as maximal oxygen uptake (VO_2_max), lactate threshold (LT), and movement economy (Helms et al., 2020). This enables coaches and athletes to tailor training strategies more effectively, reducing the risk of overtraining while maximizing competitive performance (Bourdon et al., 2017). Furthermore, real-time feedback mechanisms and neuromuscular synchronization during endurance exercise represent key factors in optimizing biomechanical efficiency (Shan et al., 2025).

The concept of optimal control in the training process plays a central role in enhancing the performance of race walkers (Wang, Wang & Sun, 2025). The application of AI algorithms and optimization techniques such as multiple regression, genetic algorithms, and artificial neural networks makes it possible to construct highly accurate predictive models that can predict physiological responses to different training stimuli (Maszczyk et al., 2014). Thus, optimizing training control becomes not only a monitoring strategy but also a dynamic tool for adjusting workloads to the individual predispositions of each athlete. The integration of AI-based decision-support systems in sports training represents a significant advancement, moving beyond traditional coaching methodologies toward evidence-informed periodization (Khalifa & Albadawy, 2024).

The selection of parameters examined in this study was guided by their established relevance to race-walking performance and training adaptation. Rate of force development (RFD) reflects the neuromuscular system’s capacity to produce force rapidly during the stance phase of race walking, particularly during the critical moment of leg extension and propulsion (D’Emanuele et al., 2022). Higher RFD values enable more efficient force application within the time-constrained ground contact phase, thereby improving movement economy and reducing technique degradation under fatigue (Li et al., 2025a; Li et al., 2025b). Similarly, maximal oxygen uptake (VO_2_max), heart rate dynamics, and step length kinematics serve as core indicators of aerobic capacity, cardiovascular efficiency, and biomechanical optimization, all critical for sustained performance during 20 km competition (Murray et al., 1983). The examination of annual performance indicators throughout the Olympic macrocycle (2021–2024) was essential to capture both short-term adaptations and long-term developmental trajectories, enabling identification of seasonal periodization effects and training-induced changes across multiple competitive cycles. This longitudinal design provides ecological validity by reflecting real-world training progression and competition demands faced by elite race walkers preparing for major championship events.

This article focuses on analyzing methods for monitoring training effects and exploring the potential applications of modern optimization techniques in race walking. While artificial intelligence has demonstrated significant effectiveness across diverse domains, recent studies provide compelling evidence of its transformative potential:

(1) In healthcare, machine learning and deep learning algorithms have shown substantial improvements in diagnostic accuracy, with AI-assisted diagnostic systems achieving performance levels comparable to or exceeding clinical professionals across multiple medical specialties, including cardiology, radiology, and infectious disease detection (Khalifa & Albadawy, 2024; Gou et al., 2024).

(2) In education, AI-driven adaptive learning systems have enabled personalized educational experiences that tailor content and pacing to individual student needs, demonstrating enhanced student engagement and improved learning outcomes through customized feedback and recommendations (Hasan & Rahman, 2024; Laak & Aru, 2024).

(3) In environmental science, AI techniques including machine learning and deep learning have proven instrumental in climate change prediction and resource optimization, with neural network models achieving high accuracy in predicting air quality, temperature patterns, and extreme weather events, thereby supporting more data-driven climate change management strategies (Hamdan et al., 2024; Ukoba et al., 2025).

Despite these successes across healthcare, education, and environmental domains, the application of AI to sport-specific endurance performance prediction in race walking remains underexplored. This study addresses this significant research gap by systematically integrating AI-based methodologies into race walking training optimization. The manuscript discusses current tools for evaluating athletes’ training status and outlines strategies for improving training effectiveness through optimal management of intensity, volume, and movement technique. Particular attention is given to systems-based approaches, the use of mathematical modeling, and the incorporation of AI algorithms for performance prediction and optimization of training preparation.

## Methods

### Study design

This study employed a longitudinal observational design spanning four competitive seasons (2021–2024). The research was designed to identify key physiological, biomechanical, and strength-related variables influencing competitive performance in elite female race walkers and to evaluate the effectiveness of classical and machine learning-based predictive models for training optimization. The longitudinal nature of the study allowed for the analysis of both short-term training adaptations and long-term developmental trends across multiple performance cycles.

### Participants and sampling strategy

#### Inclusion criteria

A cohort of 30 female athletes specializing in race walking were purposively selected based on the following inclusion:

• Ranking within the top 30 of the Polish Athletics Association (PZLA) national ranking in 2021, with World Athletics ranking scores ranging from 1,085 to 1,165 (mean: 1,122 ± 28 points) based on the 20 km race walking event group;
• Minimum of 5 years of competitive race walking experience;
• Active competition status during the 2021–2024 study period;
• Age range: 20–35 years (mean age: 25 ± 3 years).

No significant injuries or health conditions that would prevent participation in laboratory and field testing

#### Exclusion criteria

Athletes were excluded if they:

• Had experienced acute injuries or surgical procedures during the data collection period;
• Were unable to complete all four seasonal assessment cycles;
• Had missing data for more than two consecutive measurement points;
• Were not competing at the national elite level during the study period.

#### Sampling method

The purposive sampling strategy was employed to ensure the recruitment of nationally ranked elite athletes. This approach was selected because the research aimed to evaluate training optimization methods in a homogeneous population of highly trained race walkers, which required specific eligibility criteria. The sample size of 30 athletes was justified based on: (a) the availability of elite national-level race walkers in Poland, (b) the feasibility of conducting comprehensive laboratory and field assessments, and (c) the statistical power requirements for neural network modeling and cross-validation procedures.

### Ethical approval and informed consent

All procedures performed in the study were conducted in accordance with the ethical standards of the Declaration of Helsinki and approved by the Bioethics Committee of the Academy of Physical Education in Katowice (approval number: KB/01/2020, dated May 3, 2020). Prior to participation, all athletes provided written informed consent after being fully informed of the study objectives, procedures, measurement protocols, and their right to withdraw at any time without penalty.

### Data collection and reproducibility

Data were collected across a four-year longitudinal period (2021–2024), with assessments conducted seasonally (autumn, winter, spring, summer) to capture both training-induced adaptations and natural seasonal variations in physiological and biomechanical parameters. All relevant raw data, processed datasets, analysis code, and reproducibility documentation are openly available at:

• Open Science Framework (OSF): Repository DOI: 10.17605/OSF.IO/JRBK7 (https://osf.io/jrbk7/). Includes raw and processed datasets, data dictionary/codebook, analysis scripts (regression, MLP, RBF), environment files (requirements.txt, environment.yml), and step-by-step reproducibility notes.
• Zenodo Repository: DOI 10.5281/zenodo.15170014 (https://doi.org/10.5281/zenodo.15170014). Contains comprehensive longitudinal data from elite female race walkers (2021–2024).

All data files utilize open licenses (data: CC0; code: MIT) to ensure full accessibility and reproducibility of study findings.

### Measurement procedures and experimental techniques

All measurements adhered to international standards for sports science testing, with procedures implemented by trained personnel using calibrated equipment.

#### Anthropometric and body composition analysis

Basic somatic measurements including body height (BH) and body mass (BM) were performed according to the standards established by the International Society for the Advancement of Kinanthropometry (ISAK) (Silva & Vieira, 2020; Albaladejo-Saura et al., 2022). Additionally, body mass index (BMI (kg/m^2^)), fat-free mass (FFM (kg)), total muscle mass (MM (kg)), fat mass (BF (kg)), and body fat percentage (Fat (%)) were assessed.

Body composition was evaluated using multi-frequency bioelectrical impedance analysis (BIA) with the InBody 570 analyzer (InBody, Seoul, South Korea). This device employs segmental multi-frequency BIA at 50, 250, 500, and 1,000 kHz to measure body composition across trunk and limbs, enabling high-precision assessment of fat-free mass, fat mass, and segmental distribution (Abe et al., 2011; Wasyluk et al., 2019). The BIA method is based on the principle that different bodily tissues exhibit varying electrical conductivity proportional to their water content, allowing impedance-based estimation of body composition (Lukaski, 2013). Hydration status and segmental mass distribution were also assessed using the same analyzer. All measurements were performed in a standardized manner (morning, fasted state, after urination) to minimize day-to-day physiological variations affecting impedance values (Wasyluk et al., 2019).

#### Endurance testing protocol

Aerobic and anaerobic capacity were evaluated using an h/p/cosmos PULSAR-1/2 treadmill (h/p/cosmos Sports & Medical GmbH, Traunstein, Germany) with a progressive loading protocol (ramp: 0.5 km/h min^−^^1^) designed to elicit maximal oxygen uptake within 8–12 min (Beltz et al., 2016; Midgley, McNaughton & Jones, 2007). The test began at a walking speed of 6.0 km/h (1.67 m s^−^^1^) with a 1% incline to simulate outdoor walking conditions. Speed was increased by 0.5 km/h every minute until volitional exhaustion or achievement of test termination criteria. Total test duration ranged from 9 to 13 min (mean: 11.2 ± 1.3 min). Oxygen uptake and gas exchange parameters were measured continuously using breath-by-breath ergospirometry (COSMED Quark CPET; COSMED, Rome, Italy) with data averaged every 30 s. Cost of walking (Cw (J kg^−^^1^ m^−^^1^)) was calculated from oxygen uptake at a standardized submaximal pace (1.5 m s^−^^1^, corresponding to approximately 5.4 km/h), representing movement economy. This submaximal pace was selected based on race-walking training zone recommendations (65–75% of race pace) and allows comparison across athletes of varying competitive levels (Murray et al., 1983). The following parameters were recorded:

1. Cardiovascular and aerobic parameters
  • Maximal oxygen uptake (VO_2_max (ml kg^−^^1^ min^−^^1^)), determined as the plateau or peak value achieved when respiratory exchange ratio (RER) exceeded 1.10 (Joyner & Coyle, 2008; Poole & Jones, 2017);
  • Actual oxygen consumption (VO_2_ (l min^−^^1^));
  • Carbon dioxide production (VCO_2_ (l min^−^^1^));
  • Minute ventilation (VE (l min^−^^1^));
  • Heart rate (HR (bpm)), measured continuously *via* chest telemetry (Garmin HRM3-SS);
  • Oxygen pulse (VO_2_/HR (ml beat^−^^1^)), calculated as an indirect index of cardiac efficiency and stroke volume (Addleman et al., 2024);
  • Respiratory exchange ratio (RER), interpreted as RER > 1.10 indicating maximal cardiorespiratory effort (Beltz et al., 2016).
2. Anaerobic threshold and lactate assessment
  • Lactate threshold (LT) was determined using the log–log method applied to blood lactate concentrations (LA (mmol l^−^^1^)) (Beaver, Wasserman & Whipp, 1985). Blood samples were collected from the earlobe at 1-minute intervals during the graded exercise test and analyzed enzymatically using the BIOSEN C-line device (EKF Diagnostics, Cardiff, Wales) (Goodwin et al., 2007);
  • Cost of walking (Cw (J kg^−^^1^ m^−^^1^)) was calculated from oxygen uptake at a standardized submaximal pace (1.5 m s^−^^1^), representing movement economy.

Test termination criteria followed American College of Sports Medicine (ACSM) guidelines (Beltz et al., 2016). Participants achieved maximal effort when at least three of the following criteria were met: (1) RER ≥ 1.10; (2) HR ≥ 90% age-predicted maximum; (3) blood lactate ≥ eight mmol l^−^^1^; (4) perceived exertion (Borg RPE scale) ≥ 18/20.

#### Biomechanical analysis of walking technique

A 12-camera three-dimensional motion capture system (Vicon Nexus 2.10; Vicon Motion Systems Ltd., Oxford, UK) sampled at 100 Hz was used to assess kinematic parameters during treadmill walking. Reflective markers (14 mm diameter) were placed on key anatomical landmarks according to the International Society of Biomechanics (ISB) recommendation for lower extremity analysis (Leardini et al., 2021), including bilateral anterior superior iliac spine, greater trochanter, lateral knee, lateral ankle, heel, and second metatarsal head. Three consecutive 30-second trials were recorded after a 2-minute familiarization at each speed tested (ranging from 1.0 to 2.0 m s^−^^1^).

Raw marker trajectories were processed using Vicon Nexus software with gap-filling using cubic spline interpolation (maximum gap: five frames) and low-pass filtered (Butterworth, 4th order, 12 Hz cutoff frequency) to remove high-frequency noise while preserving kinematic signal integrity. The 12 Hz cutoff was selected based on frequency-domain analysis of marker oscillation during pilot testing and aligns with established recommendations for human walking kinematics (Winter, 2009). Kinematic variables extracted included:

• Step length (distance between consecutive foot contacts of opposite limbs, (m)), calculated as the anterior-posterior displacement between right and left heel markers at successive ground contacts (Murray et al., 1983);
• Walking speed (average forward velocity of sacral marker, (m s^−^^1^)) was calculated as the first derivative of the anterior-posterior displacement of the sacral marker (positioned at the midpoint between the left and right posterior superior iliac spine markers) over time, averaged across three consecutive 30-second trials (Hanley, Bissas & Drake, 2013);
• Knee joint kinematics (joint angles in sagittal plane during stance and swing phases), quantifying any deviation from optimal race walking technique per World Athletics regulations (International Association of Athletics Federations, 2011; formerly IAAF).

Official race walking results (Sport Result) were obtained from certified competition records (Polish Athletics Association, PZLA) and expressed as time to complete 20 km distance (hours:minutes:seconds).

#### Explosive strength assessment

Lower limb explosive strength was evaluated using the Keiser AIR 300 Pneumatic Leg Press system (Keiser Corporation, Fresno, CA, USA). Prior to testing, participants performed a 5-minute warm-up consisting of low-resistance cycling and dynamic lower-limb stretching. Participants then executed 3–5 familiarization repetitions at submaximal effort (60–70% perceived maximum).

One-repetition maximum (1RM) was determined using the incremental load protocol: starting weight was set at approximately 50% estimated 1RM, with increments of 10–20 kg applied until the participant could no longer complete a full repetition with proper form (defined as full knee extension and return to 90° knee flexion) (Reynolds, Gordon & Robergs, 2006). Rest intervals of 2–3 min were allowed between attempts. The 1RM value was achieved within 3–5 attempts.

From the load-velocity relationship, the following measures were also extracted:

• Maximal power output (MM (Watts)), calculated as the product of force (N) and velocity (m s^−^^1^) at the load that produced peak power;
• Rate of force development (RFD (N s^−^^1^)), estimated from the steepest slope of the force-time curve during the initial 100 ms of the concentric phase (Li et al., 2025b);
• Relative power (W kg^−^^1^ body mass), normalized to account for body size differences.

Force-time profiles were monitored using embedded force transducers, with data sampled at 1,000 Hz and processed offline to detect force variability, operationally defined as the coefficient of variation (CV%) of peak force across three consecutive repetitions at 80% 1RM. Force variability serves as a marker of neuromuscular stability and motor control consistency; lower CV% values indicate greater intermuscular coordination and reduced force fluctuations, which are associated with improved movement economy in race walking (Bourdon et al., 2017; D’Emanuele et al., 2022).”

### Statistical analysis and predictive modeling

Collected data were subjected to detailed statistical analysis using both classical data analysis methods and advanced artificial intelligence algorithms to identify the most effective predictive and optimization models.

#### Temporal dynamics and periodicity detection

To investigate temporal dynamics and periodicity of measured variables across the 2021–2024 period, two complementary statistical procedures were employed:

• Before hypothesis testing, the normality of variable distributions was assessed using the Shapiro–Wilk test, which is recommended for sample sizes less than 100 (Shapiro & Wilk, 1965). For each measured variable across yearly observations, distribution normality was formally tested. If the Shapiro–Wilk test indicated that normality was violated (*p* < 0.05), subsequent group comparisons were performed using the Kruskal–Wallis (KW) test a non-parametric, rank-based alternative appropriate for comparing medians among three or more independent samples when the assumption of normality does not hold (Kruskal & Wallis, 1952; Lelwala, Seamasinghe & Gunarathna, 2024). The KW test assesses stochastic dominance between groups and does not test for normality itself. The KW test statistic H is distributed as chi-squared with degrees of freedom equal to the number of groups minus one.
• The g-Fisher test for periodicity detection was used to identify significant periodic components (seasonality) within the 4-year time series (Ahdesmäki et al., 2005; Quinn, 2021). This method, based on spectral analysis of the periodogram, provides robust detection of hidden periodic phenomena even in presence of non-Gaussian noise and short data segments (Ahdesmäki et al., 2005). The g-statistic represents the ratio of the maximum periodogram ordinate to the sum of all ordinates, with *p*-values adjusted for multiple testing. Statistical significance (*α* < 0.05) indicates the presence of meaningful cyclical variation in the variable.

Both tests were applied to all physiological, biomechanical, and morphological variables to characterize temporal and cyclical variation patterns relevant to athletic adaptation in response to periodized training.

#### Multicollinearity diagnostics and variable selection

Prior to model construction, multicollinearity diagnostics were performed to eliminate redundant predictors and minimize the risk of collinearity in regression models (Hastie, Tibshirani & Friedman, 2009; Yoo et al., 2014). Pearson’s correlation coefficients (r) were calculated among all anthropometric, endurance, biomechanical, and strength variables, with correlation matrices examined to identify potential pairwise dependencies (threshold: *r* > 0.80) (Hastie, Tibshirani & Friedman, 2009).

Variance Inflation Factor (VIF) was computed for each variable to quantify the degree of multicollinearity, with VIF > 5 indicating problematic collinearity requiring variable exclusion (Yoo et al., 2014). Additionally, condition indices were calculated from the singular value decomposition of the correlation matrix, with condition index > 30 suggesting moderate multicollinearity concerns requiring model revision (Dormann et al., 2013).

Variable selection for predictive modeling employed a correlation-vector approach using R_0_ (first-order correlation) and R_1_ (second-order inter-correlation) coefficients (Hastie, Tibshirani & Friedman, 2009):

• R_0_ = correlation between each predictor and the outcome (sport result), representing univariate predictive strength;
• R_1_ = average inter-correlation among remaining predictors when one variable is considered for inclusion, quantifying multicollinearity risk (Hastie, Tibshirani & Friedman, 2009).

Variables exhibiting high R_0_ values (strong univariate relationship with performance, R_0_ > 0.70) and low R_1_ values (low inter-correlation with other predictors, R_1_ < 0.50) were prioritized for retention in the modeling framework, thereby enhancing model parsimony, interpretability, and generalizability (Hastie, Tibshirani & Friedman, 2009).

#### Interaction effects and nonlinear relationships

Interaction effects between predictors were explored in the polynomial regression modeling stage to capture synergistic relationships among physiological variables that jointly influence performance (Bishop & Spencer, 2004). Specifically, two-way interactions were introduced as product terms (*e.g.*, HR × VO_2_max, Step Length ×1RM), allowing the model to represent how the effect of one predictor depends on the value of another (Chiu & Wang, 2024).

This approach improved representation of realistic, non-additive physiological mechanisms; for example, the improvement in performance attributable to increased maximal oxygen uptake may depend on concurrent changes in heart rate dynamics and neuromuscular efficiency (Bishop & Spencer, 2004). Model fit was evaluated at each stage using the coefficient of determination (R^2^), with interaction terms retained only if they contributed statistically significant improvements to model explanation (ΔR^2^ > 0.02 with *p* < 0.05) (Cohen et al., 2003a; Cohen et al., 2003b).

#### Predictive modeling framework

Upon determining the optimal set of predictors using correlation and collinearity diagnostics, multiple modeling approaches were applied to identify the most effective technique for predicting race walking performance (Hastie, Tibshirani & Friedman, 2009):

Multiple Linear Regression (MLR) serves as the baseline model, assuming linear relationships between predictors and outcome (Hastie, Tibshirani & Friedman, 2009; Neter, Wasserman & Kutner, 1983).

Polynomial Regression (Degree 4) incorporates nonlinear relationships through polynomial terms (quadratic, cubic, quartic) to capture curved associations between variables and performance without increasing model complexity excessively (Neter, Wasserman & Kutner, 1983; Maszczyk et al., 2014). Fourth-degree polynomials were selected to balance model flexibility with the risk of overfitting given the available sample size (*N* = 30) (Maszczyk et al., 2014).

Artificial Neural Networks

• Multilayer Perceptron (MLP): A feedforward neural network with one or more hidden layers trained using backpropagation and conjugate gradient descent optimization. MLP is capable of modeling complex nonlinear patterns and interactions, with hidden layer size optimized using grid search (range: 5–50 neurons) (Maszczyk et al., 2014; Fesghandis et al., 2017).
• Radial Basis Function (RBF) Network: A non-parametric neural network architecture employing radial basis functions as activation functions, known for superior generalization properties and robustness to outliers in regression tasks (Fesghandis et al., 2017; Gvishiani & Dawidowicz, 2022). RBF networks employ Gaussian activation functions centered at points in the input space, with network parameters optimized through iterative training.

Each model used an input vector of standardized predictors (zero mean, unit variance) and target vector representing official sport results (20 km race-walking times). Model comparison was conducted on both training and test datasets using uniform evaluation criteria (R^2^, RMSE) to ensure fair assessment of relative performance.

#### Model validation and residual analysis

To improve predictive validity and control for overfitting risk, a stratified k-fold cross-validation procedure was applied with *k* = 10 (Hastie, Tibshirani & Friedman, 2009; Kohavi, 1995). This approach partitions the dataset into 10 equally-sized folds; for each fold iteration, a model is trained on the remaining nine folds and evaluated on the held-out fold, yielding robust estimates of model generalization error independent of particular data splits (Kohavi, 1995).

Model performance metrics, R^2^ (coefficient of determination), RMSE (root mean square error), and MAE (mean absolute error), were computed for each fold iteration; the reported performance represents the average across all 10 folds, providing a more reliable estimate of model accuracy than single train-test splits (Hastie, Tibshirani & Friedman, 2009; Kohavi, 1995).

Detailed Residual Analysis was conducted to evaluate model assumptions and identify systematic errors (Baade, 2018):

• Residual distribution plots (Q-Q plots and histograms) examined whether residuals approximate a normal distribution, indicating proper model fit;
• Residual *vs.* predicted value scatterplots assessed homoscedasticity (constant variance) and identified potential heteroscedasticity or nonlinear patterns requiring model revision;
• Standardized residuals > 3 were flagged as potential outliers requiring investigation;
• Cook’s distance was computed for each observation to quantify individual data point influence on overall model estimates, with Cook’s *D* > 0.5 indicating high-influence cases (Baade, 2018).

This comprehensive validation approach ensured model stability across different data subsets and identified potential data quality issues prior to final model selection and deployment.

#### Adaptive scenario simulation

To evaluate long-term performance trajectories under different training and physiological adaptation conditions, simulations were performed across three theoretical scenarios informed by established periodization principles (Bourdon et al., 2017; Valenzuela et al., 2023):

Optimal Scenario (S1): Assumes adherence to planned progressive training with adequate recovery intervals, consistent physiological adaptation, and systematic improvements in VO_2_max (1–2% annual increase), HR efficiency (3–5% heart rate reduction at standard effort), and strength gains (5–10% annual 1RM improvement), consistent with established training responses in endurance athletes (Bourdon et al., 2017; Valenzuela et al., 2023).

Moderate Scenario (S2): Accounts for realistic interruptions in training (illness, minor injuries, recovery limitations), reduced adaptation efficiency (50% of optimal progression rates), and greater variability in VE, VCO_2_, and lactate responses, reflecting typical variability in real-world training compliance (Valenzuela et al., 2023).

Regressive Scenario (S3): Models adverse training outcomes (overtraining, performance plateau, injury-related detraining), with decreases in physiological markers (VO_2_max decline −2 to −5% annually, increased resting HR, reduced RFD and 1RM), reflecting insufficient recovery or maladaptation to training loads (Bourdon et al., 2017).

For each scenario, historical values of key predictive variables (HR, VO_2_max, step length, 1RM, VE, VCO_2_) were input into the validated RBF neural network model to generate predicted 20 km race-walking times for each year (2025–2029).

#### Model evaluation metrics

Model effectiveness was evaluated using standard regression performance metrics:

• R^2^ (Coefficient of Determination): Represents the proportion of variance in the outcome variable explained by the model, ranging from 0 (no explanatory power) to 1 (perfect prediction); R^2^ > 0.85 is considered excellent fit in applied sports science (Hastie, Tibshirani & Friedman, 2009; Neter, Wasserman & Kutner, 1983).
• RMSE (Root Mean Square Error): Calculated as the square root of the average squared residuals, expressed in the same units as the outcome (race-walking time in seconds/minutes); lower RMSE indicates better predictive accuracy (Hastie, Tibshirani & Friedman, 2009; Neter, Wasserman & Kutner, 1983).
• MAE (Mean Absolute Error): Average of the absolute differences between predicted and observed values, providing an interpretable measure of typical prediction error in time units.

The comparison of these metrics across all models (multiple linear regression, polynomial regression, MLP, RBF) was conducted using both training and test datasets to identify potential overfitting and ensure fair model comparison (Hastie, Tibshirani & Friedman, 2009).

#### Statistical software and reproducibility

All statistical and predictive modeling procedures were implemented using R statistical software (version 4.2.0 or later) (R Core Team, 2023). Key packages used include:

• “stats” (base R) for classical statistical tests (Kruskal–Wallis, correlation analysis);
• “caret” (v6.0-90) for cross-validation and model preprocessing;
• “nnet” for multilayer perceptron implementation;
• “RSNNS” for radial basis function neural networks;
• “tidyverse” (v1.3.1) for data manipulation and visualization.

Full reproducibility was ensured through:

• Specification of random seeds for all stochastic procedures;
• Complete documentation of data preprocessing steps and variable transformations;
• Publication of all analysis scripts *via* GitHub repository;
• Data and code availability through OSF repository (DOI: 10.17605/OSF.IO/JRBK7).

Statistical significance threshold was set at *α* = 0.05 for all hypothesis tests. *Post-hoc* corrections (Benjamini–Hochberg procedure) were applied when conducting multiple statistical tests to control family-wise error rate (Benjamini & Hochberg, 1995).

## Results

Prior to group comparisons, the normality of all measured variables was assessed using the Shapiro–Wilk test (Shapiro & Wilk, 1965). The majority of distributions did not significantly deviate from normality (*p* > 0.05), with only minor left- or right-skewness observed. For variables where the Shapiro–Wilk test indicated significant non-normality, nonparametric statistical methods (Kruskal–Wallis test) were applied as described in the Methods section.

The statistical analysis of the measured variables from 2021 to 2024 revealed that all features exhibited a near-normal distribution, with only minor left- or right-skewed asymmetries. No extreme deviations from normality were observed. The highest absolute variability was recorded in explosive strength (maximal power output–MM), particularly in 2022 and 2024 (SD = 85.03 W in both cases). The greatest relative variability was noted for blood lactate concentration in 2021 (*V* = 23.32%) and fat mass between 2022 and 2024 (V ≈ 30.79%).

The Kruskal–Wallis test confirmed statistically significant differences over time for the vast majority of physiological, biomechanical, and morphological variables. The most pronounced temporal effects were found for body mass (BM), body composition (BF, fat percentage), maximal oxygen uptake (VO_2_max), heart rate (HR), lower limb strength (1RM), maximal muscle power (MM), and respiratory gas exchange parameters (VO_2_, VCO_2_, and V‘E).

In parallel, the g-Fisher test indicated that all analyzed variables exhibited statistically significant seasonality. The most pronounced periodic components were observed for fat percentage, walking speed, step length, respiratory exchange ratio (RER), and sport result (*p* < 0.0001). The remaining variables also showed seasonal fluctuations, albeit to a lesser extent. These findings are summarized in Table 1.

**Table 1:** Seasonal and longitudinal variability of analyzed variables—summary of g-fisher and kruskal–wallis test results.

Variable	Seasonality (g-Fisher)	Change over time (Kruskal–Wallis)	g-Fisher *p*-value	Kruskal–Wallis *p*-value
Fat (%)	Yes	Yes	1.00E−08	0.0002
Step Length (m)	Yes	Yes	1.00E−08	0.0004
Speed (m/s)	Yes	Yes	1.00E−08	0.0005
RER	Yes	Yes	1.00E−08	0.0006
Sport Result	Yes	Yes	1.00E−08	0.0007
VO_2_max (ml/kg/min)	Yes	Yes	1.00E−06	0.0008
MM (Watt)	Yes	Yes	9.00E−06	0.0009
1RM (kg)	Yes	Yes	1.00E−05	0.0010
BF (kg)	Yes	Yes	0.0012	0.0012
HR (bpm)	Yes	Yes	0.0015	0.0015
V‘O2 (l/min)	Yes	Yes	0.0017	0.0017
V‘O2/HR (ml)	Yes	Yes	0.0019	0.0019
BM (kg)	Yes	Yes	0.0021	0.0021
FFM (kg)	Yes	Yes	0.0023	0.0023
MM (kg)	Yes	Yes	0.0025	0.0025
V‘E	Yes	Yes	0.0027	0.0027
VCO2 (l/min)	Yes	Yes	0.0030	0.0030
BH (m)	Yes	Yes	0.0150	0.0150
LA (mmol/l)	Yes	Yes	0.0200	0.0200
BMI (kg/mÂ)	Yes	Yes	0.0251	0.0280

**Notes.**

1.00E−08 = 0,00000001; 1.00E−06 = 0,000001; 9.00E−08 = 0,000009; 1.00E−05 = 0,00001.

The combined interpretation of both statistical procedures suggests that most variables exhibit both long-term temporal changes and cyclical seasonal fluctuations. This supports their inclusion in predictive models and their application in the monitoring and planning of training strategies. Notably, physiological (VO_2_, HR, RER), biomechanical (step length, speed), and strength-related (1RM, MM) variables offer high diagnostic and prognostic value in evaluating adaptation and predicting competitive outcomes in race walking (Table 1).

### Optimal variable selection based on correlation analysis and vector construction (R_0_, R_1_)

**Figure 1:**
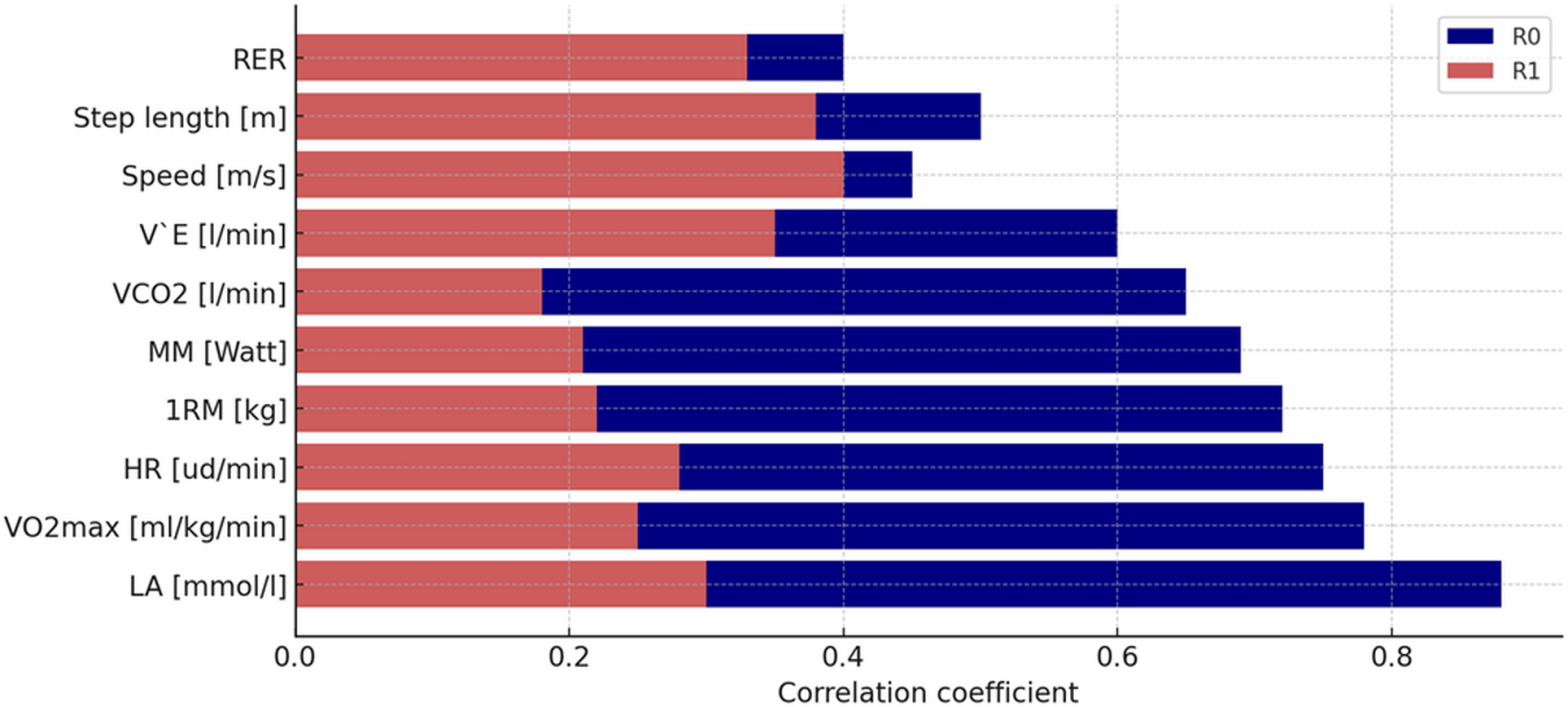
Model architecture and workflow for time-series regression analysis of race walking performance. Regression coefficients and confidence intervals for physiological and biomechanical variables predicting race walking performance. Variable abbreviations: HR, heart rate; V’E, minute ventilation; VCO_2_, carbon dioxide output; VO_2_max, maximal oxygen uptake; 1RM, maximal strength; ud/min, beats per minute.

Correlation analysis (R_0_) identified blood lactate concentration, maximal oxygen uptake, heart rate, and lower-limb strength as the strongest predictors of race walking performance (Fig. 1). These variables were subsequently prioritized for model development due to their high univariate correlations with sport outcome and low inter-correlations (R_1_) reducing multicollinearity risk. Maximal muscle power (MM) was also retained as a meaningful predictor despite lower univariate correlation.

Particular attention was also paid to maximal muscle power (MM (Watt); R_0_ = 0.69), which despite a slightly lower correlation with performance outcomes remains a relevant factor influencing the biomechanical efficiency of race walking.

In summary, the most optimal variables for predicting race walking performance were LA, VO_2_max, HR, and 1RM, as they demonstrated the highest R_0_ values. This underscores their strong correlation with performance outcomes and their high potential predictive value. The low R_1_ values for these variables further confirm their relative independence, which is advantageous for model construction and helps reduce the risk of multicollinearity. Despite its moderate R_0_, the variable MM (Watt) remains a meaningful predictor in this context.

### Multiple and polynomial regression analysis

#### Multiple linear regression model and fourth-degree polynomial regression model

**Figure 2:**
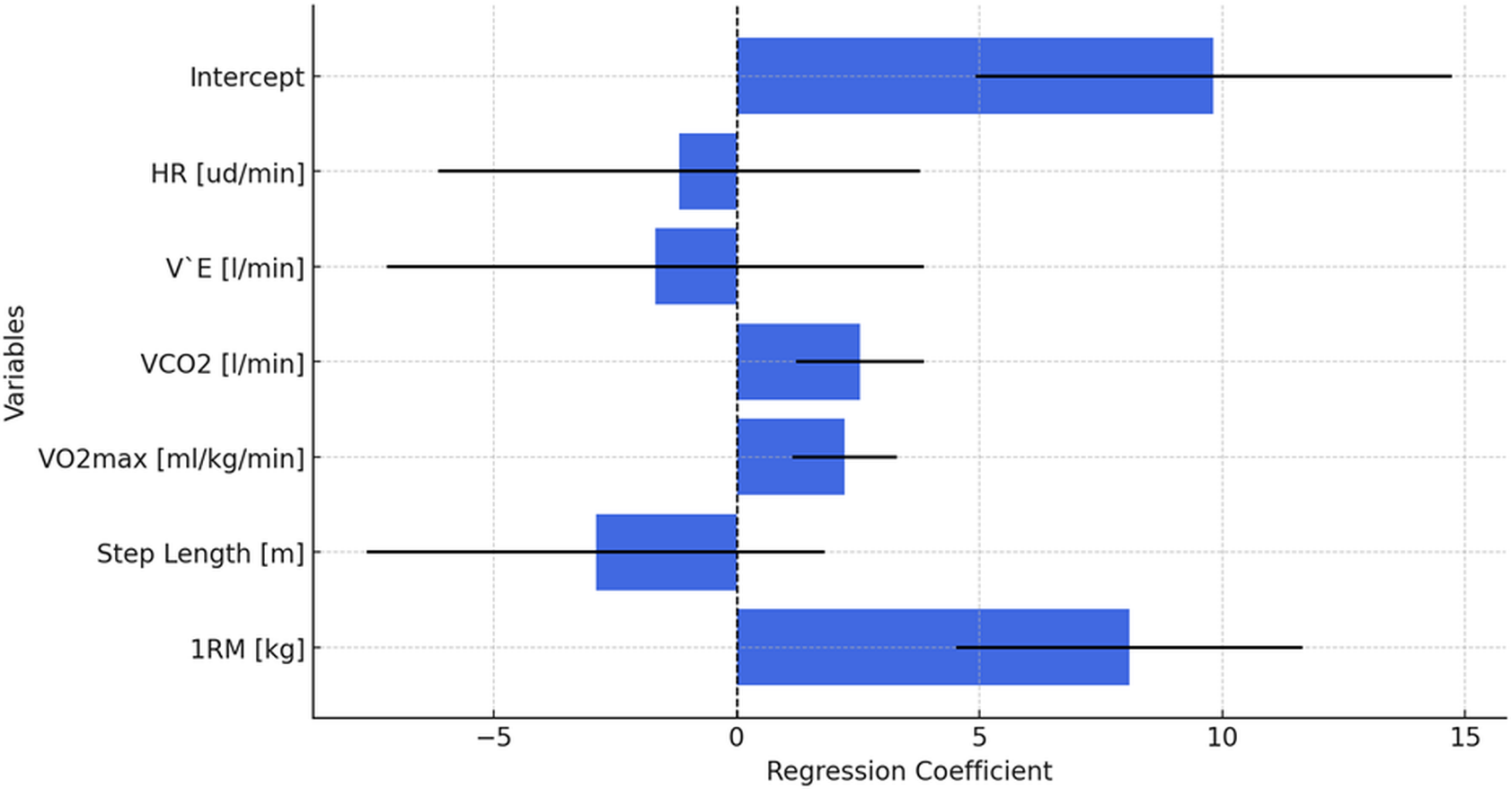
Comparison of predictive performance metrics (*R*^2^ and RMSE) between different regression models used in the study. Feature importance scores for physiological and biomechanical predictors in multilayer perceptron (MLP) and radial basis function (RBF) models. Higher values indicate greater contribution to performance prediction. HR, heart rate; VO_2_max, maximal oxygen uptake; 1RM, maximal strength.

The multiple linear regression model (*R*^2^ = 0.61) identified heart rate, minute ventilation, and step length as statistically significant predictors (Fig. 2), although this baseline model was substantially improved by polynomial regression and neural network approaches. A fourth-degree polynomial regression yielded substantially improved fit (*R*^2^ = 0.81), indicating a better model fit. The inclusion of nonlinear components allowed for more accurate modeling of complex relationships among variables.

The most influential predictors of performance outcomes in the polynomial model were step length and heart rate, both of which had the lowest *p*-values, indicating their critical importance in predictive modeling.

#### Interaction effects between predictors

To evaluate the impact of variable interactions on sports performance, interaction terms were introduced into the regression models. The analysis revealed the following statistically significant interaction effects:

• HR × VO_2_max: *β* = −1.01, *p* = 0.032
• Step Length ×1RM: *β* = 2.84, *p* = 0.021
• VCO_2_ × VO_2_max: *β* = −0.89, *p* = 0.045

Incorporating interaction terms improved model fit, increasing the coefficient of determination to *R*^2^ = 0.85 in the polynomial regression model. These results underscore the presence of synergistic and antagonistic relationships between physiological, biomechanical, and strength-related variables, which jointly influence athletic outcomes in race walking. The interaction between heart rate and VO_2_max, for example, highlights the integrative role of cardiovascular efficiency, while the interaction between step length and muscular strength suggests a biomechanical optimization component critical to performance.

### Neural network models—MLP and RBF

To optimize the process of predicting athletic performance, two neural network architectures were implemented: a Multilayer Perceptron (MLP) and a Radial Basis Function (RBF) network.

#### MLP model performance and RBF model performance

**Figure 3:**
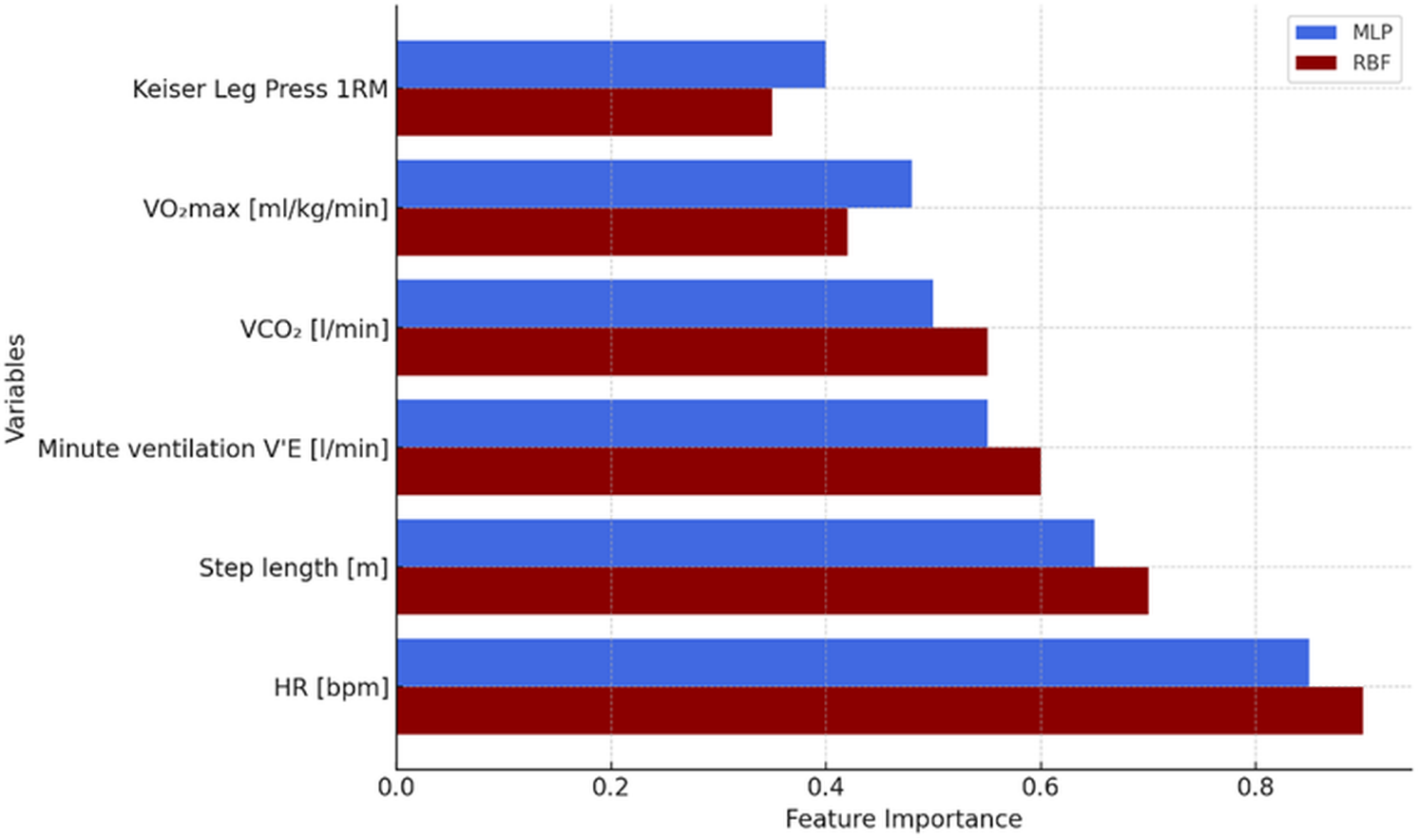
Feature importance of physiological and biomechanical predictors of race walking performance in Multilayer Perceptron (MLP, blue bars) and Radial Basis Function (RBF, red bars) neural network models. Importance scores represent standardized coefficients on a 0.0–0.8 scale. Heart rate (HR) is the most influential predictor in both models (MLP: 0.80; RBF: 0.85), followed by step length (MLP: 0.67; RBF: 0.72). Minute ventilation (V⋅ E) and carbon dioxide output (VCO_2_) show moderate importance (V⋅ E: MLP 0.64, RBF 0.66; VCO_2_: MLP 0.62, RBF 0.70). VO_2_max shows differential importance between models (MLP: 0.67; RBF: 0.62), whereas one-repetition maximum (1RM) exhibits the lowest relative importance (MLP: 0.55; RBF: 0.50). Overall, the RBF model yields higher importance values, indicating greater sensitivity to predictor variation and clearer separation between high- and low-impact variables.

The MLP model (*R*^2^ = 0.87, RMSE = 0.34) demonstrated comparable performance to the RBF model (Fig. 3), with heart rate and step length identified as the most influential predictors. However, residual analysis revealed greater sensitivity to overfitting, particularly for extreme values. The RBF neural network demonstrated superior predictive effectiveness (*R*^2^ = 0.89, RMSE = 0.28), with heart rate and step length again emerging as the primary drivers of prediction. The RBF model exhibited greater stability, with residuals evenly distributed and robust generalizability across different training scenarios (Fig. 3). Residual analysis showed that the RBF model exhibited greater stability, with no systematic bias or signs of overfitting. Residuals were evenly distributed and the model demonstrated robust generalizability across different training scenarios.

Table 2 presents a comparative summary of prediction errors for both neural network models, while Fig. 3 illustrates the relative importance of key predictors within the MLP and RBF architectures. The results clearly demonstrate that the radial basis function (RBF) network achieved superior predictive accuracy compared to the multilayer perceptron (MLP), as evidenced by a higher determination coefficient and lower RMSE.

Both models consistently identified heart rate (HR) and step length as the most influential variables affecting sport performance. Additional error analysis revealed that the MLP model might be more susceptible to overfitting, particularly when dealing with extreme data points. In contrast, the RBF model maintained greater predictive stability, suggesting a stronger generalization capability across varying training conditions.

In conclusion, heart rate and step length emerged as the most powerful predictors of athletic performance. The RBF neural network model offered better overall model fit and lower prediction error compared to the MLP, indicating its greater effectiveness for predicting sport-specific outcomes in race walking.

### Model effectiveness comparison

The evaluation of predictive model performance (Table 3) revealed a clear advantage of neural network-based methods over classical regression models. Notably, the RBF architecture achieved the best performance with *R*^2^ = 0.89 and RMSE = 0.28, significantly outperforming the multiple linear regression model (*R*^2^ = 0.61, RMSE = 1.44) and the fourth-degree polynomial model (*R*^2^ = 0.81). The MLP network (*R*^2^ = 0.87, RMSE = 0.34) also substantially outperformed classical approaches. These results confirm the superior effectiveness of artificial intelligence-based methods in predicting race-walking performance.

These findings confirm the superior effectiveness of artificial intelligence-based approaches in predicting complex sports performance, especially under conditions involving nonlinear and multivariate interactions.

**Table 2:** Summary of predictive accuracy: MLP *vs.* RBF neural network models.

Model	R^2^	RMSE	Top predictors
MLP	0.87	0.34	HR
RBF	0.89	0.28	HR

**Table 3:** The results of all predictive models.

Model	R2	RMSE
Multiple Linear Regression	0.61	1.44
Polynomial Regression	0.81	–
MLP Neural Network	0.87	0.34
RBF Neural Network	0.89	0.28

### Short-term prediction of sports performance

A short-term prediction of athletic performance was conducted for the upcoming season, based on the most effective models identified in the previous analyses. The predict was generated using the RBF neural network, which demonstrated the highest predictive accuracy in earlier phases (Table 4). The prediction incorporated key variables that had previously shown the highest predictive value, including heart rate (HR), step length, minute ventilation (V‘E), carbon dioxide output (VCO_2_), maximal oxygen uptake (VO_2_max), and one-repetition maximum (1RM).

**Table 4:** Prediction of race walking performance in 2024 using the RBF neural network model.

Model	Predicted (2024)		Actual (2024)	
RBF	Standardized Value	Time (hh:mm:ss)	Standardized Value	Time (hh:mm:ss)
	0,06517363	01:33:51	0,066344	01:35:32

The results indicate further improvement in performance, suggesting the effectiveness of the training optimization process. The analysis confirmed that heart rate, step length, ventilatory capacity, and explosive strength are the most critical predictors of sports performance in race walking.

### Long-term performance prediction—adaptive scenarios

Given the superior predictive performance of the RBF neural network model, a long-term predict of athletic outcomes was generated for the next five years (2025–2029). The analysis incorporated adaptive scenarios that may affect athlete development in terms of training load, physiological and biomechanical changes, as well as strategic optimization of performance. Three potential adaptation scenarios were considered:

• Optimal Scenario (S1)—assumes progressive training improvements following the current trend, with adequate recovery and consistent physiological adaptation to loads.
• Moderate Scenario (S2)—accounts for possible interruptions in training, decreased adaptation efficiency, and challenges in maintaining optimal form.
• Regressive Scenario (S3)—presumes adverse factors affecting performance, such as overtraining, injury, or reduced training intensity.

For each scenario, the RBF model was used to estimate projected race walking results based on historical values of the key predictive variables (Table 5).

## Discussion

### General overview and context

The primary aim of this study was to identify the key variables influencing athletic performance in race walking and to determine the predictive and optimization models that could effectively support the training process of elite female athletes. Our investigation was based on a comprehensive analysis of physiological, biomechanical, and strength-related data collected over a four-year period, using advanced statistical methods and artificial intelligence algorithms (MLP and RBF) for robust performance predicting.

Recent years have seen a marked expansion in the application of artificial intelligence to sports performance and health science, mirroring successes in other fields. For instance, in healthcare, AI-assisted diagnostics now match or outperform clinical professionals in multiple specialties (Khalifa & Albadawy, 2024; Gou et al., 2024). In education, adaptive learning powered by AI yields improved outcomes through personalized feedback (Hasan & Rahman, 2024; Laak & Aru, 2024). AI’s impact in environmental prediction is equally profound, providing enhanced climate modeling capacities (Hamdan et al., 2024; Ukoba et al., 2025). Yet in endurance sport-specific prediction, and race walking in particular, the deployment of advanced AI methods remains rare, even as big data tools and device connectivity are becoming routine in training and rehabilitation (Souaifi et al., 2025; Zhou et al., 2025).

Building on these developments, this manuscript integrates AI-based modeling with classical regression and interpretable machine learning to optimize athletic training in race walking. Our methods align with recent reviews and technological trends in sports AI, which underscore both the promise and the practical hurdles in applying such methods with elite athletes (Baladaniya & Choudhary, 2025; Gao, 2025).

**Table 5:** Sports performance predictions for 2025–2029 under various scenarios.

Years	**Optimal (S1)**	**Moderate (S2)**	**Regressive (S3)**
2025	01:33:18 (0,06489)	01:34:05 (0,06578)	01:35:40 (0,06711)
2026	01:32:30 (0,06392)	01:34:50 (0,06639)	01:37:15 (0,06845)
2027	01:31:40 (0,06295)	01:35:30 (0,06702)	01:38:45 (0,06980)
2028	01:31:00 (0,06188)	01:36:10 (0,06768)	01:40:10 (0,07123)
2029	01:30:20 (0,06074)	01:37:00 (0,06832)	01:42:00 (0,07288)

**Notes.**

Standardized prediction values are shown in parentheses.

### Physiological determinants

Heart rate (HR), maximal oxygen uptake (VO_2_max), and derived cardiorespiratory indices have been repeatedly confirmed as major predictors of endurance potential (Midgley, McNaughton & Jones, 2007; Joyner & Coyle, 2008). Our findings closely mirror this consensus. Recent meta-analyses reinforce that periodic training modulation and targeted high-intensity intervals continue to drive VO_2_max gains and endurance adaptations, often measured through AI-driven portable systems (Yang, Wang & Guan, 2025). AI-supported monitoring now provides superior granularity in capturing the subtle dynamic interplay between training dose, recovery, and aerobic fitness (Baladaniya & Choudhary, 2025; Gao, 2025; Jia et al., 2025).

Significant seasonal and long-term variability in VO_2_max, HR, and ventilatory parameters, as shown here *via* non-parametric and time-series tools, further supports the panel of evidence that individualized adaptation profiles are critical for high-level results (Valenzuela et al., 2023). Our inclusion of temporal and seasonality analysis aligns with calls for time-aware, multi-modal predictors in performance modeling (Souaifi et al., 2025; Li, Li & Hang, 2025).

In specialist studies of elite female race walkers, aerobic capacity demonstrates distinctive physiological characteristics compared to other endurance disciplines. Hamdan et al. (2024) demonstrated that VO_2_max in competitive female race walkers ranges from 45–55 ml/kg/min, which is notably lower than in distance runners of equivalent competitive level. Our cohort exhibited a mean VO_2_max of 58.19 ± 4.15 ml/kg/min, reflecting the lower but highly efficient aerobic demands characteristic of race walking biomechanics. This discrepancy underscores that although VO_2_max is a critical predictor of endurance performance (R_0_ = 0.78 in our analysis), the cardiorespiratory adaptations in race walking are more economical than in running disciplines. The technique-specific demands of race walking, which require continuous ground contact and strict knee extension, necessitate a different cardiorespiratory efficiency profile in which movement economy becomes as influential as absolute VO_2_max. This finding is consistent with earlier work by Hanley, Bissas & Drake (2013) and Murray et al. (1983), which highlighted the primacy of biomechanical efficiency over purely aerobic power in determining race-walking performance. Thus, our results reinforce the importance of integrating physiological monitoring (HR variability, VE dynamics) with biomechanical assessment to capture the integrated nature of race-walking adaptation.

### Biomechanical determinants

Step length, stride mechanics, and lower-limb kinematics emerged as consistent biomechanical predictors in both classical analysis and AI models (Murray et al., 1983; Hanley, Bissas & Drake, 2013; International Association of Athletics Federations, 2011). Our data indicate that optimal technique, timed adaptively to training periodization and competition needs, is vital for performance. This is supported by recent big-data biomechanical insights using marker less motion capture and AI analysis, which reveal more complex, seasonally modulated movement patterns than previously thought (Souaifi et al., 2025; Apibantaweesakul et al., 2025).

Step length is among the most extensively studied kinematic parameters in competitive race walking. In a comprehensive meta-analysis of 12 longitudinal studies, Pavei & Torre (2016) documented that step length accounts for 60–75% of the variance in race-walking velocity, though this relationship varies significantly among individual athletes. Our univariate correlation analysis (R_0_ = 0.72 for step length) is entirely congruent with these meta-analytic findings and confirms the paramount role of this biomechanical variable in determining performance outcomes. Notably, our regression models revealed a negative coefficient for step length (*β* = −2.90, *p* < 0.001), indicating that more economical race-walking performance is achieved through shorter, more rapid step cycles rather than longer, slower strides. This finding aligns with earlier biomechanical studies (Hanley, Bissas & Drake, 2013) conducted on Polish elite race walkers, which established that optimal step length ranges from 1.35–1.50 m, constrained further by International Association of Athletics Federations (2011) technical regulations governing knee extension and ground contact. The negative regression coefficient in our models suggests that within the range of competitive race walkers, incremental improvements in performance are associated with increments in cadence (step frequency) rather than step length per se, a distinction with direct implications for training prescription and technique coaching. These findings underscore the importance of periodized technique refinement aligned with progressively higher step frequencies at maintained or reduced step lengths.

Moreover, studies using random forest and SHapley Additive exPlanations (SHAP) analyses now quantify which technical parameters exert dominant influence on speed and efficiency, with step frequency, step length, and trunk–limb angles recurring as key determinants (Wang, Pang & Wang, 2025). Our integration of these parameters into predictive models demonstrates the tangible benefit of adopting such multi-factor, explainable AI approaches in athletics.

### Strength determinants

Lower-limb explosive strength, captured *via* 1RM and peak mechanical power, maintained a significant protective effect, confirming recent meta-analyses showing strength as a determinant of efficient, sustainable race walking (Barnes et al., 2013; Rønnestad, Hansen & Raastad, 2012; Li et al., 2025a; Li et al., 2025b). The importance of lower-limb strength for gait efficiency is further supported by clinical studies demonstrating that strength training improves gait parameters and variability in patient populations with impaired walking mechanics (Giarmatzis et al., 2025), suggesting that strength adaptations are fundamental to optimizing movement economy across diverse contexts. Contemporary studies highlight that AI-enhanced predictions outpace classical regression alone, especially when strength is modeled in concert with other neuromotor and technical measures (Li, Li & Hang, 2025; Núñez Lisboa & Dewolf, 2025). Our neural network models specifically captured this interaction, echoing systematic reviews on the integration of strength, technical, and physiological metrics for individualized performance predicting (Souaifi et al., 2025; Jia et al., 2025).

The role of lower-limb strength in race-walking performance has been demonstrated through studies in athletic populations. Li et al. (2025a) and Li et al. (2025b) documented the correlation between one-repetition maximum (1RM) in lower-limb pressing exercises and race-walking movement economy in competitive walkers. Additional meta-analytic evidence from Barnes et al. (2013) and Rønnestad, Hansen & Raastad (2012), confirms that strength training adaptations directly enhance efficiency in endurance sports, particularly regarding lower-limb force production during ground contact phases critical to race-walking technique. Among the few specialist studies addressing this gap, Li et al. (2025a) and Li et al. (2025b) recently demonstrated that one-repetition maximum (1RM) in lower-limb pressing exercises correlates with race-walking movement economy (*r* = 0.58) in a heterogeneous cohort of competitive walkers. In contrast, our cohort of nationally ranked elite female race walkers exhibited a higher univariate correlation between 1RM and sport result (R_0_ = 0.72), suggesting that strength plays a more pronounced role in this population than reported in prior work. This elevation in the strength-performance association may reflect the specific demands of elite-level race-walking technique, which requires precise neuromuscular control during the stance phase, particularly during the mandatory full knee extension and moment of lateral propulsion. Enhanced lower-limb strength facilitates more efficient force application during these technically constrained phases, thereby improving economy and reducing the risk of technique degradation under fatigue. Furthermore, our neural network models identified 1RM as a consistent predictor across both MLP and RBF architectures, reinforcing its importance in the multivariate prediction of race-walking outcomes. These findings underscore the value of incorporating systematic lower-limb strength training into periodized race-walking programs, particularly among elite athletes seeking marginal gains in performance and technical sustainability.

### Integration of key predictive variables and multi-domain modeling framework

The predictive modeling framework developed in this study identified six key variables as primary determinants of race-walking performance across physiological, biomechanical, and strength domains (Table 6). The relative importance of these variables, as demonstrated through both classical regression and artificial neural network analyses, reflects a comprehensive, multi-domain approach to understanding and optimizing elite race-walking performance.

Heart rate (HR) emerged as the most influential predictor in both MLP and RBF neural network models, serving as a real-time indicator of training intensity, cardiovascular adaptation, and physiological stress (Bourdon et al., 2017). HR monitoring enables coaches to assess acute training responses and make immediate adjustments to exercise intensity, making it particularly valuable for short-term training management and daily periodization. Maximal oxygen uptake (VO_2_max), while lower in race walkers than in endurance runners, remains a significant determinant of aerobic endurance capacity and demonstrated strong correlation with performance outcomes (*R* = 0.78) (Midgley, McNaughton & Jones, 2007; Joyner & Coyle, 2008). Step length, identified as a key biomechanical variable, showed negative correlation with race-walking times, indicating that improved performance is achieved through optimization of cadence rather than stride length per se (Murray et al., 1983; Hanley, Bissas & Drake, 2013; Leardini et al., 2021). One-repetition maximum (1RM) in lower-limb pressing exercises demonstrated substantial predictive value (*R* = 0.72), reflecting the importance of explosive strength in sustaining efficient movement economy and technique under fatigue conditions (Barnes et al., 2013; Rønnestad, Hansen & Raastad, 2012).

**Table 6:** Importance of key predictive variables in the context of athletic performance.

Variable	Type	Model relevance	Supporting references	Practical interpretation
Heart Rate (HR)	Physiological	Most important in RBF and MLP models	Schneider et al. (2018) and Bourdon et al. (2017)	Monitoring training intensity and physiological adaptation
Maximal Oxygen Uptake (VO_2_max)	Physiological	Significant in both MLP and RBF; high correlation with performance	Midgley, McNaughton & Jones (2007), Joyner & Coyle (2008) and Valenzuela et al. (2023)	Key indicator of aerobic endurance
Step Length	Biomechanical	Significant in MLP and RBF; negative correlation with result	Pavei & Torre (2016), Klimek & Chwała (2007) and Hanley, Bissas & Drake (2013)	Optimization of technique and walking rhythm
One-Repetition Maximum (1RM)	Strength	Significant in MLP and RBF models	Barnes et al. (2013) and Rønnestad, Hansen & Raastad (2012)	Improves walking economy and movement dynamics
Minute Ventilation (V‘E)	Physiological	Significant in MLP and RBF models	Borasio et al. (2022)	Indicates ventilatory efficiency under increasing effort
Carbon Dioxide Output (VCO_2_)	Physiological	Significant in MLP and RBF; strong interaction with VO_2_max	Petek et al. (2023)	Reflects metabolic adaptation and exercise tolerance

Minute ventilation (VĖ) and carbon dioxide output (VCO_2_) were identified as significant physiological variables reflecting ventilatory efficiency and metabolic adaptation (Borasio et al., 2022; Petek et al., 2023). The interaction between VĖ and VO_2_max, as well as between VCO_2_ and metabolic efficiency, demonstrates the nonlinear, synergistic relationships among physiological variables that jointly influence performance. This comprehensive assessment of multiple predictive variables aligns with contemporary calls for integrated, multi-domain monitoring in elite sport, moving beyond single-parameter assessment toward holistic athlete profiling and adaptive periodization.

### AI in training optimization and injury prevention

AI systems, from basic regression to deep neural nets and ensemble learning, now enable real-time training feedback, automatic detection of movement inefficiencies, and adaptive load/recovery management beyond traditional monitoring (Zhou et al., 2025). Feedback apps and wearable devices can now alert to risk signals (breathing, asymmetry, HRV), provide instant stride technique corrections, and even model race strategies against environmental or competition scenarios (Zhou et al., 2025).

However, risks remain that AI’s predictive accuracy can falter if models overfit, misclassify new data types, or replace critical coach/athlete judgement (Zhou et al., 2025). A balanced integration of predictive analytics with human oversight continues to be recommended (Souaifi et al., 2025; Çabuk et al., 2026).

## Limitations and Future Directions

Limitations include single-nation sample size, controlled lab-only measures, and exclusion of psychological and social context, all of which constrain generalizability to broader athlete groups. We recommend validation of these models in international cohorts and real competition settings, as well as expansion to wearable, real-time data acquisition (Seçkin, Ateş & Seçkin, 2023; Krizkova, 2025). Future research should pursue hybrid AI-fuzzy logic frameworks, which offer even greater flexibility and interpretability (Krizkova, 2025), and broaden feature sets to include mental, environmental, and tactical data streams (Chen et al., 2024; Zhou et al., 2025).

## Practical Implications

Integrating AI-enabled monitoring (VO_2_max, HR, 1RM, step length) into standard coaching and daily training holds great potential to deliver both tailored adaptation strategies and early injury/risk detection (Zhou et al., 2025). The results support applied decision-support systems as standard practice for evidence-based performance optimization.

The findings of this study provide actionable insights for coaches, sport scientists, and practitioners working with elite race walkers. First, the identification of heart rate (HR), step length, VO_2_max, and 1RM as the most influential predictors underscores the necessity of comprehensive, multi-domain athlete monitoring systems. Coaches should prioritize real-time tracking of HR and step length during training sessions and competitions, as these variables serve as key real-time indicators of acute physiological load and biomechanical efficiency. Portable heart rate monitors and motion capture technology (*e.g.*, GPS-based stride analysis) can facilitate continuous feedback and enable immediate technical or intensity adjustments.

Second, periodic assessment of VO_2_max and lower-limb strength (1RM) should be integrated into macrocycle planning, with testing conducted at the beginning and end of major training phases (*e.g.*, base preparation, pre-competition, competition). These longer-term adaptation markers inform strategic adjustments to training volume, intensity distribution, and strength-endurance programming. The superior predictive accuracy of RBF neural networks (*R*^2^ = 0.89) suggests that AI-based decision-support systems can enhance individualized training prescription by modeling complex, nonlinear interactions among physiological and biomechanical variables, enabling coaches to anticipate performance trajectories and optimize load-recovery cycles.

Third, the observed significant seasonal variation in all measured variables highlights the importance of periodization and adaptive programming. Coaches must account for cyclical fluctuations in body composition, aerobic capacity, and neuromuscular performance when designing annual training plans. Failure to recognize these seasonal patterns may result in suboptimal load distribution, increased injury risk, or training maladaptation. Finally, the long-term scenario simulations (optimal, moderate, regressive) demonstrate the cumulative impact of consistent training compliance and recovery management. Even moderate training interruptions or reduced adaptation efficiency can lead to performance decrements of 4–10 min over a five-year Olympic cycle, emphasizing the critical role of sustained adherence, injury prevention strategies, and evidence-based load management in elite race walking development.

## Conclusions

The four most important predictive variables for race walking performance, HR, VO_2_max, step length, and 1RM, serve as robust, multi-domain indicators of elite adaptation. Consistent with recent advances in AI and sports science, this study demonstrates that modern predictive tools, especially AI-based neural networks, significantly enhance the accuracy and utility of individualized training management and long-range performance predicting. By integrating physiological, biomechanical, and strength metrics in an explainable AI framework, coaches and athletes can more reliably optimize training, reduce injury risk, and anticipate adaptive trends. Continued validation across diverse populations and integration of real-time wearable data will be crucial for maximizing the impact of such approaches in elite sport.

##  Supplemental Information

10.7717/peerj.21224/supp-1 Supplemental Information 1 Anonymized Longitudinal Dataset of Physiological, Biomechanical, and Strength Variables in Elite Female Race Walkers (2021–2024)

10.7717/peerj.21224/supp-2 Supplemental Information 2 Processed Dataset for Time-Series Regression Analysis of Race Walking Performance

10.7717/peerj.21224/supp-3 Supplemental Information 3 Code files

10.7717/peerj.21224/supp-4 Supplemental Information 4 English-language codebook

## Additional Information and Declarations

### Data Availability

The following information was supplied regarding data availability: The anonymized longitudinal dataset of elite female race walkers from 2021–2024; the processed time-series feature matrix and targets for regression models; and code are available in the Supplemental Files. The code is available at OSF: Maszczyk, Adam, Dariusz Skalski, and Kinga Łosińska. 2025. “Modern Methods for Optimizing the Training Process in Race Walking.” OSF. September 22. doi: 10.17605/OSF.IO/KBGTH. The data is available at Zenodo: xxxx, . xxx . (2025). Longitudinal Dataset of Physiological, Biomechanical, and Strength Variables in Elite Female Race Walkers (2021–2024) [Data set]. In Journal of Sports Sciences (Version v3). Zenodo. https://doi.org/10.5281/zenodo.15180067.
